# Controlled trial of a workplace sales ban on sugar-sweetened beverages

**DOI:** 10.1017/S1368980023001386

**Published:** 2023-10

**Authors:** Jamey M Schmidt, Elissa S Epel, Laurie M Jacobs, Ashley E Mason, Bethany Parrett, Amanda M Pickett, Leyla M Mousli, Laura A Schmidt

**Affiliations:** 1 Sutter Health California Pacific Medical Center Research Institute, San Francisco, CA, USA; 2 Department of Psychiatry, University of California at San Francisco, San Francisco, CA, USA; 3 Philip R Lee Institute for Health Policy Studies, University of California at San Francisco, San Francisco, CA, USA; 4 Department of Humanities and Social Sciences, University of California at San Francisco, San Francisco, CA 94143, USA

**Keywords:** Obesity prevention, Workplace interventions, Sugar-sweetened beverages, Diabetes prevention, Food environments

## Abstract

**Objective:**

To examine the effectiveness of a workplace sugar-sweetened beverage (SSB) sales ban on reducing SSB consumption in employees, including those with cardiometabolic disease risk factors.

**Design:**

A controlled trial of ethnically diverse, full-time employees who consumed SSB heavily (sales ban *n* 315; control *n* 342). Outcomes included standardised measures of change in SSB consumption in the workplace (primary) and at home between baseline and 6 months post-sales ban.

**Setting:**

Sutter Health, a large non-profit healthcare delivery system in Northern California.

**Participants:**

Full-time employees at Sutter Health screened for heavy SSB consumption.

**Results:**

Participants were 66·1 % non-White. On average, participants consumed 34·7 ounces (about 1 litre) of SSB per d, and the majority had an elevated baseline BMI (mean = 29·5). In adjusted regression analyses, those exposed to a workplace SSB sales ban for 6 months consumed 2·7 (95 % CI –4·9, –0·5) fewer ounces of SSB per d while at work, and 4·3 (95 % CI –8·4, –0·2) fewer total ounces per d, compared to controls. Sales ban participants with an elevated BMI or waist circumference had greater post-intervention reductions in workplace SSB consumption.

**Conclusions:**

Workplace sales bans can reduce SSB consumption in ethnically diverse employee populations, including those at higher risk for cardiometabolic disease.

Sugar-sweetened beverages (SSB; e.g. sodas, sports drinks and ‘fruit’ drinks) are the single largest source of added sugar in the diet^(1)^. Ubiquitous in workplace cafeterias and vending machines, SSB are hyperpalatable and energy-dense and produce limited satiety^(2)^. Recent meta-analyses find that SSB consumption is a significant risk factor for obesity, abdominal adiposity, hypertension, diabetes and CHD^(3–6)^. SSB are also a key driver of health disparities in obesity and cardiometabolic disease^(1,7)^.

Adults spend most of their waking hours at work, making the workplace an efficient environment for implementing policy interventions that reduce SSB consumption. Yet currently, many workplaces contribute to the problem. Food and beverages purchased in the workplace are generally higher in added sugars than those available at home^(8–10)^, and SSB are the most frequently purchased food item at work^(11)^. If workplace policies to reduce SSB consumption are proven effective, employers are likely to be incentivised to adopt them, given high healthcare and productivity costs for obesity and cardiometabolic diseases^(12,13)^.

The workplace SSB sales ban is an emerging non-governmental policy for reducing the health risks of heavy SSB consumption^(13)^. Despite limited research on their effectiveness, many health systems, health departments, city governments, schools and universities in the USA, UK, Canada and Australia have spontaneously begun experimenting with sales bans^(14–19)^. Following strategies to achieve tobacco-free workplaces, a sales ban entails the removal of SSB from all workplace sales outlets, replacing them with non-sugary beverage options, while still allowing employees to bring SSB from home. Consistent with behavioural economics and research on food environments, sales bans could help to ‘nudge’ employees towards healthier options^(20,21)^, while reducing environmental cues and triggers that drive hedonic consumption^(22)^.

Workplace sales bans are a mainstay for tobacco prevention, with well-documented effectiveness^(23–27)^. Yet sales bans have, so far, received limited attention in the literature on public health strategies for mitigating potentially harmful food environments. So far, studies are small-scale and limited to observational designs, with no controlled trials^(28–32)^. In our uncontrolled pilot study of an SSB sales ban, heavy-drinking employees experienced significant reductions in SSB consumption after 6 months of exposure, with disproportionately large declines for employees with obesity. Decreases in SSB consumption also correlated with reductions in waist circumference and insulin resistance^(32)^.

This article reports on the first controlled trial of SSB sales ban in the workplace. Heavy-drinking employees at eight Northern California hospital campuses were exposed to an SSB sales ban *v*. no sales ban (the control condition). We hypothesised that the sales ban would reduce employee SSB consumption after 6 months of exposure, and that its effects would be more pronounced in employees with risk factors for cardiometabolic disease, including elevated BMI and elevated waist circumference.

## Methods

This controlled trial took place during 2018–2020 in eight hospital campuses (*n* 5 sales ban and *n* 3 controls). The sample consisted of 657 full-time employees who consumed SSB heavily at baseline (at least three SSB per week), with 315 employees in the sales ban condition and 342 in the control condition. Sites belonged to Sutter Health, a not-for-profit, integrated healthcare system in Northern California. We recruited hospitals that: (1) had geographic proximity to one of two fieldwork teams, (2) had hospital leaders willing to participate and (3) were balanced in organisational size across conditions (measured by the number of full-time equivalent employees). Assignments of sites to each condition were selected in cooperation with hospital leaders and were balanced by size.

The trial was originally registered on clinicaltrials.gov (NCT03431051) and Open Science Framework (https://osf.io/cxgbk/) with waist circumference at 12 months as the primary outcome and SSB consumption a secondary outcome. In-person data collection for this trial was closed in March 2020 due to the COVID-19 pandemic, preventing waist circumference assessments for sixty-nine participants at 6 months and 207 at 12 months. Trial registration and power calculations, which had assumed a 12-month period for observing effects on waist circumference, were revised following published guidelines for pandemic trial closures^(33,34)^. SSB consumption at work became the primary outcome, with changes expected after 6 months of exposure to a sales ban based on results from the pilot study^(32)^. To explore the effects of the intervention on employees with cardiometabolic disease risk factors, we stratified the sample on normal *v*. elevated BMI and waist circumference measured at baseline, where anthropometric data were complete.

### Sales ban intervention

A workplace sales ban calls for the removal of SSB from all workplace sales venues, including cafeterias, vending machines, food courts and other retail outlets. SSB are defined as beverages with added sugars, including sodas, sports drinks, ‘fruit’ drinks, sweetened coffees, and teas. Employees and visitors can still bring in and consume SSB purchased outside the workplace.

In the five sales ban sites recruited for this study, the study team worked closely with hospital leaders, vendors, and suppliers to consistently adopt and implement a new procurement policy that eliminated SSB while promoting substitutes without added sugars (e.g. artificially sweetened beverages, bottled water and unsweetened tea). Notification of the sales ban was posted in the hospital cafeterias 2 weeks prior to implementation. Random audits by study staff of all food and beverage outlets were conducted monthly, resulting in approximately 500 location visits. Overall compliance was adequate with 82·5 % of visits finding no SSB. Most violations were due to confusion by wholesale purchasers about the sugar content of beverages and were referred to the director of Food and Nutrition Services for prompt removal. Three control hospital campuses continued to sell SSB as usual.

### Participant recruitment

The sample was comprised of employees who reported heavy SSB consumption at baseline. Inclusion criteria included: (1) consumed 24 oz. or more of SSB per week, (2) worked full time at a participating Sutter Health campus and (3) not pregnant. Participants were recruited through letters, flyers, screen savers, staff meetings, huddles, cafeteria outreach, and town hall meetings and screened for eligibility in person by study staff prior to the baseline assessment.

### Procedures

To facilitate fieldwork, recruitment was staggered across three cohorts of employees from 2–3 hospitals each, between January 2018 and November 2019. Participants were compensated with gifts valued between $100 and $200. Institutional review boards at Sutter Health and the University of California, San Francisco, approved all procedures.

At the baseline and follow-up assessments, demographics and dietary measures were collected in-person or remotely using REDCap. At baseline, trained staff collected anthropometric measures (height, weight and waist circumference) in private locations at the employee’s work site. Dietary measures collected after the COVID-19 closure were conducted exclusively online, although 86 % (*n* 496) of participants had completed the final assessment prior to closure. Because hospitals remained open and continued food and beverage service during the pandemic, those remaining participants in the intervention condition were still exposed to sales ban, except for < 1 % of those shifted to remote work.

**Fig. 1:**
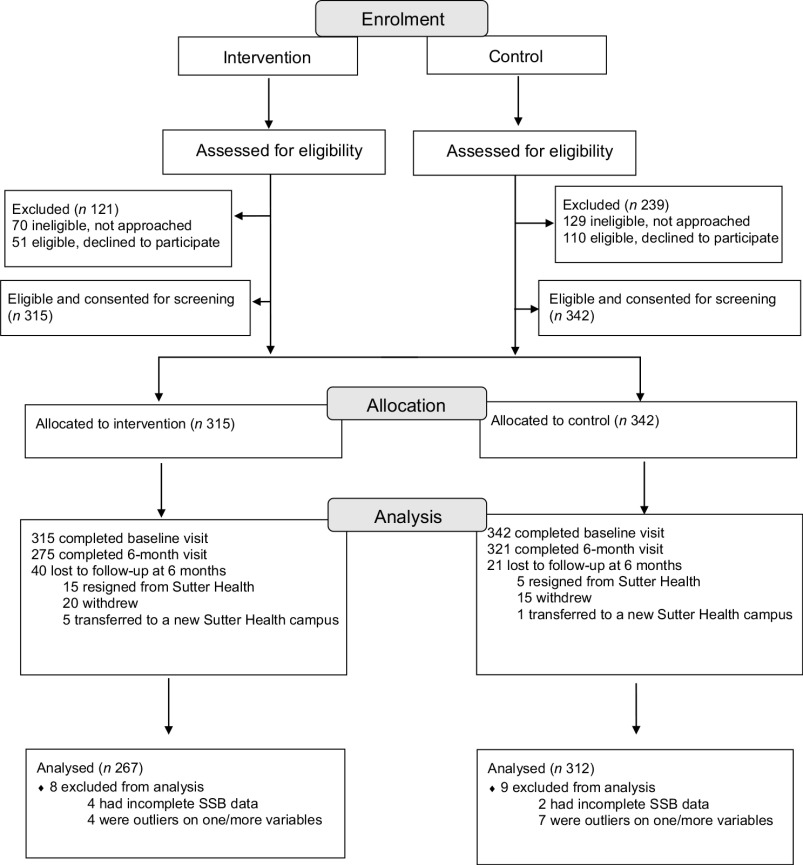
Participant flow through the trial of SSB sales ban. SSB, sugar-sweetened beverage

The retention rate was 90·7 % (*n* 596; Fig. 1) and was similar across the sales ban (87·3 %; *n* 275) and control (93·9 %; *n* 321) conditions. Loss to follow-up was primarily due to staff resignations from Sutter Health or participants transferring to a non-participating Sutter Health campus.

### Dietary measures

#### Sugar-sweetened beverage consumption

We assessed SSB consumption using the validated, fifteen-item Beverage Intake Questionnaire, the BEV-Q^(35)^. We made minor adaptations to the BEV-Q, tested in a pilot study^(32)^, to capture beverages consumed at the workplace and while not at the workplace. This allowed us to assess: (1) SSB consumption while at work (primary outcome), (2) SSB consumption outside of work (to assess the potential for compensation or increased consumption outside the workplace in sales ban participants) and (3) total SSB consumption (both in the workplace and outside the workplace combined).

The BEV-Q asks about the type and frequency of consuming specific types of beverages on a typical day: regular/non-diet soda, diet soda, 100 % fruit juice, fruit drinks (e.g. lemonade and smoothies), sports or energy drinks, sweetened coffee, or tea drinks (e.g. Arizona iced tea and Starbucks Frappuccino), and water. Daily intake was calculated for each beverage type by multiplying the frequency of intake and serving size. SSB included all regular or sugar-sweetened sodas, ‘fruit’ drinks, sports drinks, energy drinks and pre-sweetened coffee and tea drinks.

#### FFQ

We used the electronically formatted Fat/Sugar/Fruit/Vegetable Screener of Block FFQ^(34)^, a standardised and validated dietary assessment tool^(36,37)^. This instrument addresses select foods consumed over the past year, with adjustments for usual intake of low-fat/trans-fat-free or low-carbohydrate/low-sugar foods. Using validated computer algorithms, the Block produces estimates of added sugars (in sweetened cereals, soft drinks and sweets), as well as other aspects of diet composition. As a validity check on the primary outcome, we tested the association between change in SSB consumption at work (as measured by the BEV-Q) and change in total added sugars in the diet, including SSB (as measured by the Block), from baseline to follow-up, finding that these correlations were statistically significant (see online supplementary material, Supplemental 1).

### Anthropometric measures

This analysis stratified the sample on baseline cardiometabolic disease risk using the BMI and waist circumference. We measured BMI (weight in kg/height in m^2^) at baseline using a standardised stadiometer for height and a calibrated scale for weight, where a BMI ≥ 25 is considered an elevated weight that puts individuals at higher risk for cardiometabolic diseases^(38)^. All weight measurements were taken without shoes, with a hospital gown over undergarments. We measured abdominal adiposity^(39)^, as waist circumference (in cm), just above the right iliac crest at the mid-axillary line using the NHANES method^(40)^. We used a calibrated tension measuring tape (Gulick II, Country Technology) with a separate tape for each participant. Measurements were made to the nearest millimetre, measuring three times, taking the mean of the closest two measurements. Following prior studies, the distribution was dichotomised to reflect normal reference values (< 80 cm for women and < 94 cm for men) and elevated (≥ 80 cm for women and ≥ 94 cm for men)^(41)^.

### Statistical analysis

All analyses were performed in StataSE version 15.1^(42)^. First, we computed means and standard deviations, or percentages, as appropriate for each variable, for participant demographic factors, predictor variables and outcome variables at baseline.

Second, we characterised each BMI and waist circumference at baseline in relation to four categories of SSB consumption (≤ 12 oz. per d, 12·1–24 oz. per d, 24·1–36 oz. per d and > 36 oz. per d). We performed paired sample *t* tests between each category of SSB consumption to ascertain which groups differed.

Third, a series of regression analyses tested the hypothesis that, compared to the control condition, participation in the sales ban condition would predict larger reductions in SSB consumption. We conducted regression analyses predicting the three SSB consumption outcomes (workplace consumption, consumption outside the workplace and total consumption). Models of consumption outside the workplace were used to assess the potential for compensatory intake in the sales ban condition.

All regression models were adjusted for sex, race/ethnicity, baseline BMI and baseline SSB consumption. Our primary analysis focused on between group (sales ban condition *v*. control condition) changes in SSB consumption from baseline to follow-up. Because the study condition variable is binary (sales ban = 1; control = 0), coefficients for condition are interpretable as the additional oz. reduction per d attributable to being in the sales ban condition. Last, we repeated the regression analyses stratified on normal *v*. elevated BMI and waist circumference.

## Results

### Baseline characteristics

We recruited a total of 657 participants. Participants were predominantly female (73·9 %), with a mean age of 40·5 years (sd 10·8) at baseline (Table 1). The ethnic representation was broad, with 33·9 % of the sample identifying as non-Hispanic White, 9·3 % as Black/African American, 24·1 % as Latino, 26·2 % as Asian American, and 6·5 % as other or unknown. Income and education were evenly distributed, with most participants having at least some college and earning over $60 000 per year. Several health-related risks were present in the sample. The mean BMI was 29·5 (sd 6·9), which is above the clinical threshold for overweight (BMI ≥ 25) and close to the threshold for obesity (BMI ≥ 30). The mean waist circumference was 98·7 cm (sd 15·6), and mean baseline consumption of SSB was 34·7 oz. (sd 35·2) per d (about 1 litre).

**Table 1:** Baseline sample characteristics

	Full sample			Sales ban			Control		
	%	Mean	sd	%	Mean	sd	%	Mean	sd
Sex (*n* 656) (%)*									
Female	73·9			68·8			78·7		
Male	26·1			31·2			21·4		
Missing (*n*)	1			1			0		
Mean age (*n* 654) (sd)		40·5	10·8		41·1	11·3		40·0	10·3
Missing (*n*)	3			2			1		
Race/ethnicity (*n* 657) (%)									
Non-Hispanic White	33·9			34·3			33·6		
Black/African American	9·3			9·9			8·8		
Hispanic/Latino	24·1			21·0			26·9		
Asian/Asian American	26·2			28·9			23·7		
Other or Unknown	6·5			6·0			7·0		
Income (*n* 638) (%)									
< $30 000	2·5			2·7			2·4		
$30 000–60 000	21·3			20·5			22·0		
$60 000–90 000	21·6			21·9			21·4		
$90 000–120 000	16·0			14·6			17·3		
$120 000–150 000	12·1			12·6			11·6		
$150 000+	26·5			27·8			25·3		
Missing (*n*)	19			6			13		
Education (*n* 646) (%)†									
High school or lower	7·4			7·5			7·4		
Some college/tech school	33·0			27·0			38·5		
Associates degree	17·5			18·6			16·6		
Bachelor’s degree	26·9			31·8			22·4		
Master’s degree or higher	15·2			15·3			15·1		
Missing (*n*)	11			4			7		
Total SSB consumption (oz./d) (*n* 638)		34·7	35·2		32·4	34·5		36·9	35·7
Missing (n)	19			9			10		
BMI (*n* 655)		29·5	6·9		29·2	6·7		29·6	6·7
Missing (*n*)	2			1			1		
Waist circumference (cm) (*n* 655)		98·7	15·6		98·1	15·6		99·3	15·5
Missing (*n*)	2			1			1		

* *P* < 0·004.

† *P* < 0·02, for contrasts between sales ban and control conditions, %s may not total 100 due to rounding.

### BMI and waist circumference by SSB consumption

The daily total volume of SSB consumption was associated with greater cardiometabolic disease risk status at baseline (Table 2). Study participants who consumed over 36 oz. of SSB per d had significantly higher BMI (31·6, sd 6·6) than those with lower levels of consumption (*P* < 0·01 for all comparisons). The mean waist circumference (104·2 cm, sd 15·7) was also highest among participants who consumed more than 36 oz. of SSB per d.

**Table 2:** Baseline BMI and waist circumference at four different levels of total SSB consumption

	≤ 12 oz. per d (*n* 173)		12·1–24 oz. per d (*n* 156)		24·1–36 oz. per d (*n* 98)		> 36 oz. per d (*n* 211)	
	Mean	sd	Mean	sd	Mean	sd	Mean	sd
Baseline BMI	27·3	5·5	28·6	7·1	29·3	6·5*	31·6	6·6†,‡,§
Baseline waist circumference (cm)	94·1	13·8	96·6	15·2	97·9	14·5||	104·2	15·7†,‡,¶

SSB, sugar-sweetened beverage.

* Significantly different from 12 oz. group (*P* < 0·02).

† Significantly different from 12 oz. group (*P* < 0·001).

‡ Significantly different from 12–24 oz. group (*P* < 0·001).

§ Significantly different from 24–36 oz. group (*P* < 0·003).

|| Significantly different from 12 oz. group (*P* < 0·04).

¶ Significantly different from 24–36 oz. group (*P* < 0·001).

### Effects of sales ban *v.* control on change in SSB consumption

On average, all participants, both in the sales ban and control conditions, experienced decreases in SSB consumption between baseline and the 6-month follow-up (workplace consumption mean change: –5·7 oz., sd 18·4; total consumption mean change: –11·5 oz., sd 33·0). Table 3 presents adjusted regression results indicating that, compared to participants in the control condition, those in the sales ban condition had significantly greater decreases in workplace consumption and total SSB consumption (in the workplace and outside the workplace combined). On average, daily workplace consumption declined by 2·7 oz. more in the sales ban condition than the control condition (*P* = 0·02; Model 1). Total daily consumption (both at the workplace and outside the workplace) decreased, on average, by 4·3 oz. more than in sales ban than the control condition (*P* = 0·04; Model 3). The sales ban and control conditions did not significantly differ in changes in daily SSB consumption outside the workplace (see online supplementary material, Supplemental 1). Unadjusted analyses (see online supplementary material, Supplemental 2) showed that without accounting for demographic factors, these effects were not statistically significant. This is likely due to the strong and statistically significant associations between SSB consumption and BMI, as well as other baseline differences (Table 1).

**Table 3:** Adjusted regression models predicting change in SSB consumption (oz. per d) from baseline to follow-up

	Model 1, SSB workplace consumption (*n* 577)		Model 2, SSB consumption outside the workplace (*n* 573)		Model 3, total daily SSB consumption (*n* 576)	
Variable	Coefficient	95 % CI	Coefficient	95 % CI	Coefficient	95 % CI
Condition	−2·7	−4·9, –0·5	−1·2	−3·6, 1·3	−4·3	−8·4, –0·2
Sales ban (1)						
Control (0)						
BMI at baseline	−0·2	−0·4, –0·0	−0·2	−0·4, 0·5	−0·4	−0·7, –0·1
SSB consumption at baseline	−0·3	−0·3, –0·3	−0·3	−0·4, –0·3	−0·7	−0·7, –0·6
Sex (male)	1·9	−0·6, 4·4	1·2	−1·6, 4·0	2·9	−1·8, 7·5
Race/ethnicity (*v*. non-Hispanic White)						
Black/African American	4·7	0·4, 8·9	5·0	0·2, 9·8	8·7	0·9, 16·6
Hispanic/Latino	1·1	−1·8, 4·0	−0·3	−3·5, 2·9	1·0	−4·3, 6·3
Asian/Asian American	0·2	−2·7, 3·0	−1·9	−5·1, 1·3	−1·1	−6·4, 4·2
Other or Unknown	1·3	−4·0, 6·7	−2·1	−8·0, 3·9	−0·6	−10·5, 9·3

SSB, sugar-sweetened beverage.

### Effects of sales ban *v.* control stratified by BMI and waist circumference status

**Fig. 2:**
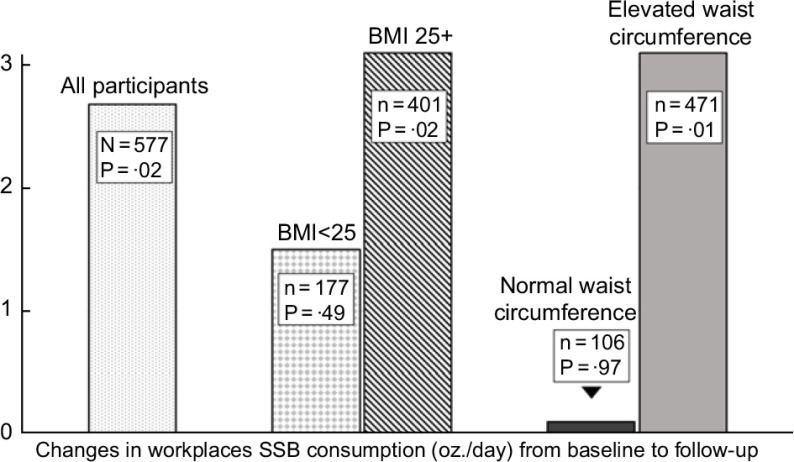
Reductions in SSB workplace consumption (oz./d) for all participants and for participants stratified by normal *v*. elevated BMI and waist circumference. Note: elevated waist circumference: >80 cm for women and >94 cm for men. SSB, sugar-sweetened beverage

Figure 2 plots the intervention’s adjusted regression coefficients reflecting changes in workplace SSB consumption, stratified on BMI (elevated *v*. normal), waist circumference (elevated *v*. normal), and compared to the coefficient for all participants from Table 3 (for complete models, see online supplementary material, Supplemental 3 and 4). Participants with an elevated BMI in the sales ban condition had significantly greater reductions in workplace SSB consumption (–3·1 oz. per d, *P* = 0·02). Similarly, participants with elevated waist circumference had significantly greater reductions in workplace SSB consumption (–3·1 oz. per d, *P* = 0·01). We did not observe these patterns for participants with a normal BMI (–1·5 for BMI < 25, *P* = 0·49) or a normal waist circumference (–0·1 oz. per d, *P* = 0·97).

## Discussion

Employers have begun to experiment with workplace SSB sales bans as a strategy for preventing obesity and cardiometabolic disease. All prior studies of the effectiveness of this non-governmental policy approach to SSB reduction have been small-scale and uncontrolled, limiting the conclusions that could be drawn. Results from this controlled trial, conducted across eight large workplace sites, suggest that the sales ban is a promising public health strategy for employees who consume SSB heavily. Study participants in both the intervention and control groups experienced secular declines in SSB consumption, a finding consistent with other studies conducted in California during a similar time frame^(43)^. However, those exposed to a sales ban for 6 months reduced their SSB consumption at work by 2·7 oz. per d more than controls. Overall, those in the sales ban condition experienced a 4·3 oz. greater reduction in total consumption (in the workplace and outside the workplace combined). Moreover, the effects of the sales ban were more pronounced in high-risk subgroups of employees with elevated BMI and elevated waist circumference.

Evidence from this trial suggests that workplace sales bans are likely to be most effective by discouraging workplace consumption, with limited spillover on consumption outside the workplace. On average, employees in the sales ban condition reduced their daily SSB consumption outside the workplace by 1·2 oz. per d more than controls, but this effect was not statistically significant. Our analyses ruled out the possibility that employees might compensate for the sales ban at work by increasing their SSB and/or sugar consumption outside the workplace. Although at least one prior study of a school-based SSB sales ban documented higher sugar compensation at home^(44)^, the current trial found no such evidence in adults. Future studies should consider strategies for augmenting sales bans with social marketing and public health education campaigns that intentionally promote spillover effects outside the workplace.

Baseline characteristics of the sample lend confidence to the conclusion that sales bans can be effective for a wide range of people, including at-risk populations. Employees recruited for this study were ethnically diverse (66·1 % non-White). At baseline, a majority of the sample had an elevated BMI (mean = 29·5) and/or waist circumference (mean = 98·7 cm). Average baseline SSB consumption in this sample was 34·7 oz. or approximately 1·0 litres per d. We also found associations between the baseline level of SSB consumption and measures of adiposity: those consuming the most SSB (36+ oz. per d) had the highest average BMI and waist circumference (*P* < 0·01).

This trial was statistically powered to explore the sales ban’s differential effects on high-risk subgroups, including those with an elevated BMI and elevated waist circumference at baseline. This is important because sales bans, by necessity, impact the whole employee population, even though those already at risk are a top public health priority. One prior study of a sales ban found disproportionate benefits in employees with obesity but also examined the impact of a brief motivational intervention and did not have a control group^(32)^. The results of this controlled trial show disproportionate decreases in workplace SSB consumption for employees with an elevated BMI (–3·1 oz. per d more than controls, *P* < 0·02) and elevated waist circumference (–3·1 oz. per d than controls, *P* < 0·01). This suggests that, while sales bans touch the whole employee population, they are most effective for those already at heightened risk for cardiometabolic diseases.

### Limitations

This study has several methodological limitations. Our primary outcome, SSB consumption, is based on the study participant’s self-reported intake. Although we used validated instruments, self-report measures tend to undercount actual consumption due to memory loss and social acceptability bias. We were able to conduct validity checks of these data against respondent self-reports of dietary sugars in a food frequency instrument. Self-reports of SSB consumption correlated with objective measures of BMI and waist circumference. Another limitation is the potential for unobserved site-level variation. The sales ban was administered at the site level, and demographic analyses of the sample revealed that participant baseline characteristics varied somewhat across participants in the intervention and control conditions, specifically on race/ethnicity and BMI. Because experimental condition was assigned at the site level and the study was limited to eight sites, we were unable to use site in the models to more broadly address variation between locations. However, models controlled some variation by including demographic variables as controls collected at the individual level. Lastly, due to COVID-19 restrictions, we were not able to examine the effects of the intervention on potential changes in adiposity.

### Implications

Study findings suggest that workplace SSB sales bans may be a promising addition to the existing arsenal of public health prevention strategies for combating obesity and cardiometabolic disease. As evidence of their effectiveness accumulates, employers may be incentivised to adopt these policies, given rising healthcare costs and productivity losses due to cardiometabolic diseases for which heavy SSB consumption is a known risk factor^(12)^. Due to the power of lobbying and organised resistance by the SSB industry, governmental strategies for SSB reduction, such as soda taxes, face significant political obstacles to adoption^(12,45)^. Non-governmental policies, including sales bans, may offer a path around these critical obstacles to SSB reform. And because sales bans are already a mainstay of tobacco control, the approach is already familiar to many employers, making it more easily implemented and scaled. While systematic research is needed, anecdotal evidence suggests that the administrative burden of launching a sales ban was relatively low. Meanwhile, vendors in sales ban sites did report large declines in beverage sales, perhaps because they stocked a variety of appealing low-sugar beverage options.

SSB sales bans may also have inherent advantages for health equity promotion that should be pursued in the future studies. Current prevention strategies used in the workplace, such as the US Centers for Disease Control and Prevention’s *Diabetes Prevention Program,* involve extended coaching in diet and fitness and can be effective for weight loss^(46–48)^. However, they disproportionately benefit people with flexible time and resources, thus selecting for higher socio-economic status and more motivated employees^(49,50)^. In contrast, sales bans require little effort from individual employees and appeared to be effective at reducing SSB consumption in this study’s diverse sample.

To maximise their impact for real-world policymaking, future trials of SSB sales bans should examine outcomes other than SSB consumption, including more distal and biological measures of cardiometabolic disease (e.g. insulin sensitivity). Because effect sizes from this study are smaller than those found in a prior study^(32)^, more research in diverse workplace environments is needed to develop robust estimates of the health benefits that can be expected from launching SSB sales bans. More rigorous trial methodologies should be considered, such as those involving cluster randomisation and statistical procedures that address site-level confounders (e.g. fixed effects modelling). SSB sales ban research should also be applied to congregate settings that reach at-risk populations not currently in the labour market, such as criminal justice settings and community colleges.

## Supplementary material

For supplementary material accompanying this paper visit https://doi.org/10.1017/S1368980023001386. click here to view supplementary material
