# Supplementary material for: Controlled trial of a workplace sales ban on sugar-sweetened beverages
Source: Public Health Nutr. 2023 Jul 19;26(10):2130–8. doi: 10.1017/S1368980023001386 (PMC10564602; doi:10.1017/S1368980023001386)
Supplement: Supplementary file 1 [file S1368980023001386sup001.docx]

**SUPPLEMENTARY MATERIALS**

**Supplement 1.** Correlations between change in sugar-sweetened beverage (SSB) consumption and change in added sugars consumption in the total diet

|  | Added Sugar Change  (n=572) | p |
| --- | --- | --- |
| Change in daily SSB consumption | 0.17 | .00 |
| Change in SSBs consumed at the workplace | 0.16 | .00 |
| Change in SSBs consumed outside of work | 0.15 | .00 |

*Note.* SSB = sugar-sweetened beverage; sugar-sweetened beverage consumption as measured by the BEV-Q; added sugars consumption as measured by the Block Food Frequency Questionnaire (total dietary and beverage sugars). Changes calculated between baseline and 6-month follow-up.

**Supplement 2.** Unadjusted regression models predicting change in sugar-sweetened beverage consumption (oz. per day) from baseline to follow up

|  | Model 1  Workplace SSB consumption (n=580) | | Model 2  SSB consumption outside the place (n=575) | | | Model 3  Total daily SSB consumption (n=577) | |
| --- | --- | --- | --- | --- | --- | --- | --- |
| Variable | Coefficient | 95% CI | Coefficient | | 95% CI | Coefficient | 95% CI |
| Condition | -1.0 | -3.8, 1.8 | 0.5 | -2.5, 3.5 | | -1.4 | -6.8, 4.1 |

*Note.* SSB = sugar-sweetened beverage; two study conditions coded as: Sales Ban = 1; Control = 0.

**Supplement 3.** Full regression models predicting change between baseline and follow-up in workplace SSB consumption, stratified on elevated vs. normal BMI status

|  | Workplace consumption (oz./day), BMI<25 (n=177) | | | Workplace consumption (oz./day), BMI 25+ (n=401) | | |
| --- | --- | --- | --- | --- | --- | --- |
| Variable | Coefficient | CI | | Coefficient | CI | |
| Condition (Sales Ban) | -1.5 | -5.7, 2.7 | | -3.1 | -5.7, -0.6 | |
| BMI at baseline | 0.8 | -0.4, 2.1 | | -0.2 | -0.5, 0.0 | |
| SSB consumption at baseline | -0.3 | -0.4, -0.2 | | -0.3 | -0.3, -0.3 | |
| Sex (male) | 0.1 | -5.3, 5.5 | | 2.3 | -0.5, -5.2 | |
| Race/Ethnicity (*vs. Non-Hispanic White*) |  |  |  |  |  |  |
| Black/African American | 3.5 | -7.5, 14.5 | | 5.4 | 0.8, 10.0 | |
| Hispanic/Latino | -4.2 | -10.9, 2.6 | | 2.4 | -0.7, 5.6 | |
| Asian/Asian-American | -1.7 | -6.5, 3.2 | | 0.9 | -2.8, 4.5 | |
| Other or Unknown | -5.8 | -16.3, 4.6 | | 4.4 | -1.9, 10.6 | |

*Note.* SSB = sugar-sweetened beverage; two study conditions coded as: Sales Ban = 1; Control = 0

**Supplement 4.** Full regression models predicting change between baseline and follow-up in workplace SSB consumption, stratified on elevated vs. normal waist circumference

|  | Workplace consumption (oz./day), normal waist circumference (n=106) | | | Workplace consumption (oz./day), high risk waist circumference (n=471) | | |
| --- | --- | --- | --- | --- | --- | --- |
| Variable | Coefficient | CI | | Coefficient | CI | |
| Condition (Sales Ban) | -0.1 | -6.6, 6.3 | | -3.1 | -5.5, -0.8 | |
| BMI at baseline | 1.2 | -0.5, 2.8 | | -0.2 | -0.4, -0.0 | |
| SSB consumption at baseline | -0.2 | -0.3, -0.1 | | -0.3 | -0.3, -0.3 | |
| Sex (male) | -2.8 | -11.0, 5.3 | | 2.2 | -0.7, 5.0 | |
| Race/Ethnicity (*vs. Non-Hispanic White*) |  |  |  |  |  |  |
| Black/African American | 1.7 | -10.2, 13.6 | | 4.4 | -0.2, 9.1 | |
| Hispanic/Latino | -2.6 | -15.7, 10.6 | | 1.5 | -1.3, 4.4 | |
| Asian/Asian-American | -2.3 | -9.7, 5.2 | | 1.1 | -2.0, 4.3 | |
| Other or Unknown | -9.7 | -29.0, 9.6 | | 2.7 | -2.7, 8.1 | |

High risk waist circumference defined as >80cm for women, >94cm for men.
